# The Effectiveness of Leg- and Arm-Powered Trike Training Among Children with Impaired Walking Ability—A Pilot Study

**DOI:** 10.3390/children12030382

**Published:** 2025-03-19

**Authors:** Loredana Tschenett, Heiner Baur

**Affiliations:** Department of Physiotherapy, School of Health Professions, Bern University of Applied Sciences, Stadtbachstrasse 64, 3012 Bern, Switzerland; heiner.baur@bfh.ch

**Keywords:** bicycling, exercise, mobility limitation, walking, disabled children, cerebral palsy, meningomyelocele

## Abstract

**Background/Objectives:** The GO-TRYKE^®^ Kid (GTK^®^) is an arm- and leg-powered tricycle which, in addition to promoting strength, endurance, and coordination, aims to reactivate the central pattern generators of the spine for locomotion through cyclical movements. The present study investigated the effects of GTK^®^ training on walking ability, GTK^®^ riding performance, and health-related quality of life in children with walking disabilities. **Methods:** Nine children trained with the device twice a week for nine weeks. Short- and long-term effects on walking ability were measured using the timed up and go test (TUG) and the two-minute walk test (2MWT). GTK^®^ riding performance and health-related quality of life were compared before and after the intervention period. **Results:** While no long-term effect on walking was found, a significant short-term effect on functional walking ability was observed (*p* = 0.009). GTK^®^ riding performance improved significantly over the training period (*p* = 0.004). There were no significant changes in health-related quality of life. **Conclusions:** GTK^®^ enables children with walking disabilities to participate in cycling as part of play and sport. Further research is required to investigate its functional and participatory effects, as there is significant potential to improve physical activity and overall well-being in this population.

## 1. Introduction

Regular physical activity is known to be indispensable for physical fitness and cardiorespiratory, metabolic, and mental health [1]. The World Health Organization’s (WHO) guidelines on physical activity and sedentary behavior from 2020 recommend children and adolescents aged 5–17 years to do at least an average of 60 min of moderate-to-vigorous physical activity per day. In addition, these 60 min should include intensive physical activity at least three times a week in order to have a positive effect on health [2,3]. The WHO physical activity guidelines for children and adolescents with disabilities do not differ from those for children and adolescents without disabilities. Further disease-specific recommendations for children with cerebral palsy (CP) are provided by Verschuren et al. [4], who found a positive effect of exercising at an intensity of 60–95% of maximum heart rate at least two to three times per week. Contrary to long-standing concerns about the potential risks of cardiorespiratory exercise for children with CP, their results indicated a low incidence of injury.

The latest global data show the worrying trend that 81% of healthy adolescents do not reach the recommended level of physical activity [5]. Martin Ginis et al. [6] found that people with disabilities are 16–62% less likely to adhere to the WHO’s physical activity guidelines compared to the general population. Among children with disabilities aged 11–15 years, only an estimated 8.5–40.4% meet the physical activity guidelines, with girls being less active than boys [7]. Children living with disabilities are confronted with multiple physical, psychological, and social barriers to participate in physical activities such as play and sport [8]. It is therefore not surprising that children with physical disabilities are, on average, less physically active than their healthy peers. The physical activity of children with CP has been studied predominantly in this context [9,10]. Notably, the extent of this disparity varies across different types of disabilities and is most pronounced in children with multiple impairments, lower motor function, and advancing age [11,12].

**Figure 1:**
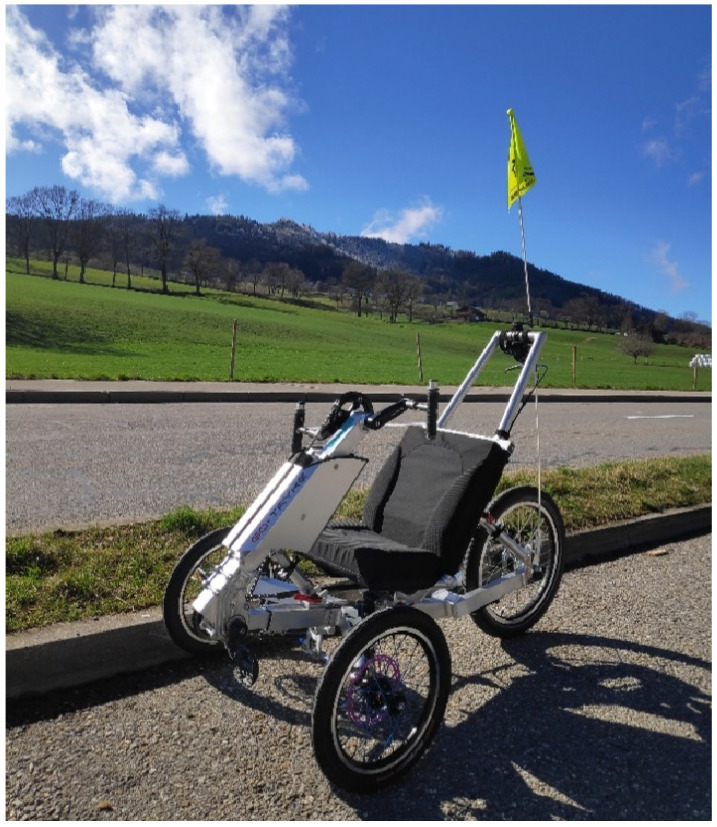
GO-TRYKE^®^ Kid device (GoByYourself, GBY^®^, Vuisternens-en-Ogoz, Switzerland).

In order to promote both the physical and mental health of children with physical disabilities, there is an urgent need to provide opportunities for these children to participate in both play and sport. These opportunities should be age-appropriate, fun, and motivating, as long-term continuation depends on the ease and enjoyment with which they are carried out [13]. A newly developed recumbent tricycle with an alternating arm- and leg-coupled drive, called GO-TRYKE^®^ Kid (GTK^®^), could be a suitable training and sports device for children with walking impairments (Figure 1). It was developed by the company GBY^®^ SA (GO-TRYKE^®^, GBY^®^ SA, Vuisternens-en-Ogoz, CH, https://www.gby.swiss/de (accessed on 15 January 2025) for people with reduced mobility. Since the tricycle is powered by the arms and legs, it facilitates an active use of the lower limbs even if the ability to achieve repetitive full cycling revolutions with the legs alone is limited. The GTK^®^ has a large seat and backrest, making it possible for people with weak torso stability to cycle. Riding a bicycle is a popular form of recreation and physical activity for children and is also a way to develop positive peer relationships [14]. Regular GTK^®^ riding, like riding a regular bicycle, could improve riding skills and contribute to better physical fitness [15,16]. This could further influence the children’s health-related quality of life through the known positive effects of regular physical activity [17]. Targeted training could be used to promote specific skills in individual areas such as cardiopulmonary endurance, strength, or coordination.

Many of the sports devices currently available for people with walking disabilities are operated with the upper extremities only and neglect the lower extremities, even though they often have residual functions. Most of the existing adapted bicycles are hand bikes. In contrast to riding a hand bike, the inclusion of the legs ensures repetitive cyclic active-assistive mobilization which, together with the effects mentioned above, can lead to a reduction in the spasticity of the lower extremities [18]. In addition, another key aspect of GTK^®^ training is that it might have an influence on walking ability. An improvement in walking would be expected, on the one hand, due to an increase in strength endurance, coordination, and short-term reduction in spasticity and, on the other hand, due to a possible reactivation of the spinal central pattern generators (CPGs) aimed for by the manufacturers of the GTK^®^. CPGs are neuronal circuits at the spinal level that are responsible for generating rhythmic movements such as breathing, walking, and swimming. They contain information on how to stimulate different motor neurons in the correct sequence and intensity and thus generate motor patterns [19,20]. It is hypothesized that in brain or spinal cord injury, undamaged central pattern-generating circuits are present below the lesion and it may be possible to generate patterned output from these regions if they are activated appropriately, either with neuromodulators, direct electrical stimulation, or sensory input [21,22,23]. Such sensory reactivation is aimed at in GTK^®^ training through the repetitive crossed reciprocal movement of the extremities, which occurs when walking.

Therefore, the aim of this study was to investigate the effects of regular GTK^®^ training sessions over nine weeks on walking ability, GTK^®^ riding performance, and health-related quality of life among children with impaired gait function due to neurological disorders. Additionally, the feasibility and usability of the GTK^®^ in practice were assessed.

## 2. Materials and Methods

### 2.1. Study Design

This was a single-arm, longitudinal, exploratory field study with two measurement dates, one at baseline and the other after the nine-week training intervention period. The measurements and training sessions took place at the Schulbildung Stiftung Rossfeld in Berne. Due to the small sample size and the resulting non-generalizability of the results, the present study did not fall under the responsibility of the country of origin’s law on human research. The intervention is an equivalent treatment alternative that is not associated with greater risks than the usual therapy content. Official ethical approval by the local authority was therefore not necessary.

### 2.2. Participants

This study included children and adolescents who had limited walking function due to neurological disorders but who were able to walk a minimum of ten meters with or without aids. Further inclusion criteria were age between 6 and 18 years and cognitive ability to comply with the study protocol, follow instructions, and communicate pain. Children and adolescents who suffered from acute health disorders, felt pain or discomfort during cycling, had severe spasticity or contractures that limited cranking, showed low compliance, or could not ride a common bicycle were excluded.

The children and adolescents who met the above criteria and their parents were verbally informed about the study and asked about their interest in participating. A written declaration of consent was then obtained, which had to be signed by both the participants and one parent or guardian.

### 2.3. Intervention

The participants trained individually with the GTK^®^ device twice a week for nine weeks. The sessions lasted between 20 and 30 min and replaced usual physiotherapy sessions. The physiotherapists normally responsible for the children and adolescents led and supervised the training sessions. They received instruction on the usage of the GTK^®^ devices and training setup before the start of the intervention phase. It was mainly trained on small asphalt paths and forest trails. In case of heavy rain, the GTK^®^ was mounted on a stationary roller trainer so that the sessions could be carried out indoors.

A central and motivating aspect of the GTK^®^ riding is the opportunity to move independently on varying terrain and at different paces. Cusick et al. [13] found that long-term adherence to training programs depends on the ease and enjoyment of participation. Standardization of the intensity of the training sessions was therefore targeted by using the OMNI scale for the subjectively perceived intensity in combination with the therapist’s observation of the participant’s fatigue (reduced cycling frequency, altered breathing, etc.) [24]. A value of 4–7 on the OMNI scale of 1–10 was aimed for, which corresponds to a moderate to vigorous training effort. The training parameters distance, mean velocity, maximum velocity, and training duration were recorded with a bike computer (Van Rysel^®^, Model BC, Lille, France).

### 2.4. Outcomes

The primary outcome investigated in the present study was walking ability. The secondary outcomes examined were GTK^®^ riding performance, health-related quality of life, the feasibility of GTK^®^ training, and the usability of the devices. Walking ability was divided into two components: functional walking ability, which was assessed using the timed up and go test (TUG), and walking endurance, which was measured with a two-minute walk test (2MWT). The TUG is a valid and reliable instrument to assess functional mobility in the pediatric population. Functional mobility includes walking and the movement transitions that contribute to walking, such as sitting up, sitting down, and turning [25]. The 2MWT, on the other hand, correlates strongly with the 6MWT in the pediatric population and can therefore effectively replace it to objectify walking endurance [26].

GTK^®^ riding performance was quantified based on the distance covered in a six-minute cycling test (6MCT) conducted on a flat indoor circuit of 60 m. In addition to the distance, this test documented the number of help interventions needed by the participants to get the device back on course or to start. Health-related quality of life was assessed with the Pediatric Quality of Life Inventory (PedsQL). This is a questionnaire consisting of 23 items, which are both child self-reported and parent proxy-reported [27]. The questions are divided into physical, social, emotional, and school functioning categories, allowing the questionnaire to be interpreted in terms of the individual dimensions. The PedsQL questionnaire was found to be a feasible, reliable, and valid instrument to assess health-related quality of life in the pediatric population with chronic conditions [28,29].

**Figure 2:**
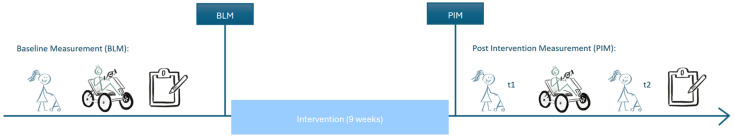
Timeline of the outcome measurements.

The outcomes were measured on two dates, at baseline and after the nine-week intervention period. The sequence of outcome measurements in the baseline measurement (BLM) was TUG, 2MWT, 6MCT, and PedsQL. In the post-intervention measurement (PIM) the walking tests were performed twice, once before the GTK^®^ cycling warm-up and test and once immediately afterward TUG, 2MWT, 6MCT, TUG, 2MWT, PedsQL (Figure 2). This made it possible to investigate both the short-term and long-term effects of GTK^®^ training on walking ability. The long-term effects were evaluated by comparing the BLM with the PIM. To evaluate the short-term effects, the walking tests of the post-intervention measurement were compared both before (PIM t1) and after an individual five- to ten-minute warm-up and the 6MCT with the GTK^®^ (PIM t2).

Feasibility of the GTK^®^ training and usability of the devices were observed and reported during and after the study. For each session, the supervising physiotherapist documented the training parameters measured with the bike computer and additional information if something special occurred. After completing the study, the physical therapists were asked to complete an online questionnaire with eight questions and a space for voluntary comments. The questions were related to the handling of the GTK^®^, the applicability in practice, and the perceived motivation of the participants (Table A3).

### 2.5. Analysis

The data were analyzed descriptively to obtain an overview of the distribution and trends. Box plots were used to visualize the data graphically. Due to the small sample size and as the data were not normally distributed, Wilcoxon tests were carried out to examine the central tendencies. To evaluate the long-term effects, the measurements of the walking tests, the GTK^®^ cycling test, and the health-related quality of life assessment at baseline (BLM) and after the intervention (PIM) were examined. To evaluate the short-term effects on walking ability, the two walking tests that were performed during the post-intervention measurement at time t1 and t2, before and after the GTK^®^ cycling, were tested. The questions answered online by the physiotherapists regarding the feasibility of the GTK^®^ training and the usability of the devices were summarized descriptively to obtain an impression of the use of the GTK^®^ in a practical therapeutic setting.

## 3. Results

Nine participants with heterogeneous diagnoses and clinical symptoms were included in this study (Table 1). There were no dropouts. Two training sessions per week were planned for all participants. The number of sessions completed depended on absences due to sickness and school-related timetable changes. All participants completed 12 to 17 training sessions during the nine-week intervention period lasting an average of 21 min (Table A1).

To evaluate the long-term effects on physical performance, the results of the baseline measurement (BLM) and post-intervention measurement (PIM) of the walking tests and the 6MCT were compared (Table 2). Both walking test results were in a very similar range at baseline and after the nine-week training intervention. The TUG results of the BLM ranged from 8 to 57 s, and the ones of the PIM ranged from 8 to 53 s, whereas the 2MWT results of the BLM ranged from 40 to 151 m, and the ones of the PIM ranged from 42 to 153 m. However, the results of the 6MCT ranged from 0 to 600 m at baseline and from 290 to 950 m post-intervention. Participants covered 260 to 590 m more in the PIM than in the BLM. Six participants required assistance during the 6MCT at baseline and one participant was not able to operate the GTK^®^ despite help-interventions. In contrast, in the post-intervention measurement, only three participants needed assistance during the test.

The results of the two walking tests performed during the post-intervention measurement immediately before (PIM t1) and after (PIM t2) the GTK^®^ riding were used to evaluate the short-term effects. All participants showed reduced TUG values in the measurements after GTK^®^ riding. On average, the TUG was reduced by 2.67 s. No consistent trend was found for the 2MWT (Table 3).

**Figure 3:**
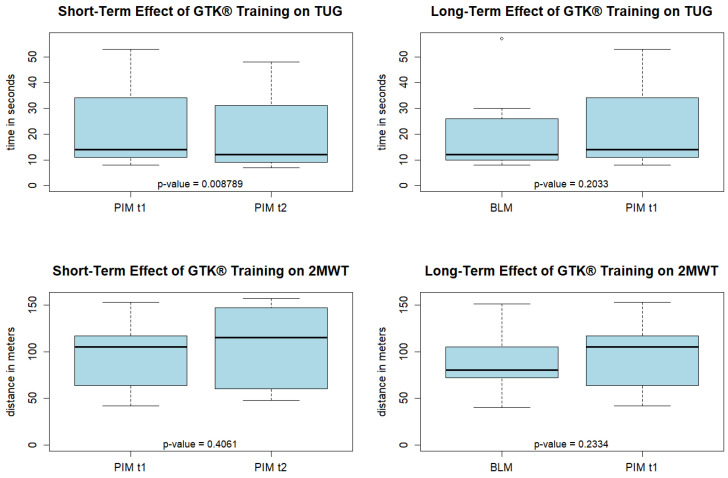
Box plots describing the short- and long-term effects of GTK^®^ riding on the walking tests. TUG: timed up and go; 2MWT: 2 min walking test; BLM: baseline measurement; PIM t1: walking tests during post-intervention measurement before GTK^®^ riding session; PIM t2: walking tests during post-intervention measurement after GTK^®^ riding session.

The short- and long-term effects of GTK^®^ training on walking ability are presented descriptively in the box plots in Figure 3. Functional walking ability measured with the TUG showed no consistent long-term trend over the intervention period (*p* = 0.2033). Although the analysis of the long-term effects on walking endurance (2MWT) showed a slight upward shift in the central trend, this was also not statistically significant (*p* = 0.233). The analysis of the short-term effects, however, showed a significant improvement in functional walking ability (*p* = 0.009) immediately after riding the GTK^®^. No significant short-term effects on walking endurance (2MWT) were observed (*p* = 0.406).

**Figure 4:**
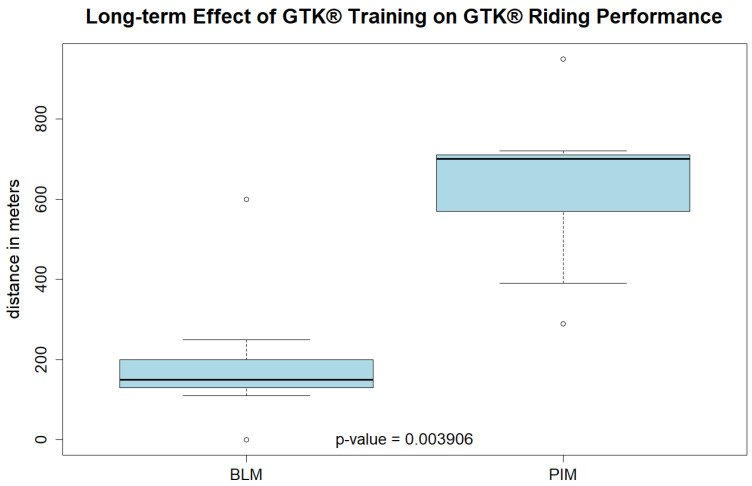
Box plots of the 6MCT (six-minute cycling test) at baseline (BLM) and at the post-intervention measurement (PIM).

A significant improvement was observed when comparing GTK^®^ riding performance (6MCT) at baseline and post-intervention (*p* = 0.004). On average the participants cycled 440 m more in the 6MCT in the post-intervention measurement than at baseline (Figure 4).

In the assessment of health-related quality of life using the PedsQL questionnaire, four dimensions of quality of life were proxy-reported (PR) and self-reported (SR): physical, emotional, social, and school functioning (Table A2). The proxy-reported assessment was conducted with the responsible social pedagogue, who is the child’s reference person at school. It was found that the total value of the results did not change significantly over the course of the training period (SR: *p* = 0.128, PR: *p* = 0.068). When looking at the individual dimensions, however, there was a significant improvement in the self-reported assessment of emotional functioning (*p* = 0.036) but no difference in the proxy-reported assessment (*p* = 0.156). No significant changes were found either in the dimension of physical, social, or school functioning.

This exploratory field study showed that regular GTK^®^ training is feasible with the selected population. The participants’ motivation for the nine-week training period was rated as moderate to high by the therapists. Most therapists who supervised the training sessions found the equipment to be user-friendly, simple, and intuitive to operate. Although there was sufficient space for the transfer from the wheelchair to the seat of the device, most of the study participants required assistance either with the transfer or with placing and securing their feet on the pedals. According to the therapists, the biggest challenge for the participants was steering, which, unlike a normal bike, works with a tilt and not a turn and requires familiarization. For further development of the device, the therapists would like the children and adolescents to be able to operate the gearshift themselves rather than it having to be operated by a second person as is currently the case. Furthermore, they would like for the device to restrict the field of vision less. Overall, the therapists see a potential use of the device in a heterogeneous user group of people with CP, spina bifida, ataxias, spinal cord injuries, and muscular dystrophies in the early stages. Severe contractures, unilateral symptoms, and limited cognitive abilities impair the use of the GTK^®^ (Table A3).

## 4. Discussion

The aim of this study was to investigate the effects of regular GTK^®^ training on walking ability, GTK^®^ riding performance, and health-related quality of life in children and adolescents with walking impairments. Furthermore, the feasibility of regular GTK^®^ training and the usability of the devices was assessed. In this study, twice-weekly GTK^®^ training for nine weeks did not have a long-term effect on functional walking ability (TUG) and walking endurance (2MWT). However, it was found that riding the GTK^®^ had a significant short-term effect on functional walking ability as measured by the TUG, with an average decrease in time of 2.67 s. This finding is consistent with the observations of the supervising physiotherapists, who noticed that the children and adolescents were able to walk more easily and fluidly after the training sessions. Several mechanisms could be responsible for these results. One reason could be the regulation of muscle tone through cycling. Previous studies have shown that passive cycling reduces reflex excitability in patients with spinal cord injury [18]. Therefore, the repetitive cyclical movement might have reduced spasticity in the participants with spasticity, and the gait pattern was less strongly characterized by spasticity for a short time after the training sessions. Another explanation could be related to the CPGs lying below the level of the lesion. Repeated cyclic reciprocal movement of the extremities could lead to sensorimotor stimulation of the CPGs via the afferents, thus influencing their activity. In this way, GTK^®^ riding would recall the spinal locomotion patterns that function without supraspinal control [23]. However, the expression of the CPGs and their influence on the population studied is unknown. It is assumed that CPGs are innate and are later shaped and refined through experience [19]. Since none of the children and adolescents studied were ever able to walk without impairment, this would mean that the CPGs could never be properly trained. Therefore, it is not known whether and how the neuronal circuits of the CPGs can be retrained in this population.

Although a significant short-term effect of GTK^®^ riding on functional walking ability was found, this must be interpreted with caution. The minimal clinically important difference (MCID) of the TUG is highly dependent on the walking ability of the subjects. For children and adolescents with CP classified as GMFCS (Gross Motor Function Classification System) level II, which corresponds to community ambulators in this study, the estimated MCID for a moderate change is 2.68 s. A minimal change was observed in children and adolescents with GMFCS level III, which corresponds to household and therapy ambulators in this study; the estimated MCID is 4.65 s, and, for moderate change, 38.60 s [32]. It can therefore not be assumed that the functional walking ability achieved a short-term clinically relevant improvement. The effects must be interpreted individually, considering the functional level of the participants. No short-term effects on walking endurance were recorded after a GTK^®^ session. This could be related to muscular fatigue after cycling.

The reason why no long-term effect of GTK^®^ training on walking ability was found may be that the activity of GTK^®^ riding differs too much from walking. Studies in people after spinal cord injuries have shown that the gravitational load during gait training and the hip joint-related afferent inputs, i.e., the hip angles, are decisive for the effectiveness of gait training [33]. During GTK^®^ riding, both the gravitational load and the hip joint angles deviate substantially from those during walking. Moreover, the additional coordination and balance aspects that are required for walking are completely neglected during GTK^®^ training. Nevertheless, these aspects have a major influence on the walking ability of the population under investigation. The results of the present study show no evidence that regular GTK^®^ training alone has a positive influence on walking ability. However, as there are indications of a short-term improvement in walking ability after GTK^®^ riding, it would be interesting to investigate a combination training of GTK^®^ riding and gait training, e.g., with GTK^®^ riding as preparation for subsequent gait training.

Riding performance with the GTK^®^ improved significantly over the nine-week training period. The participants covered 260 to 590 m more in the post-intervention measurement of the 6MCT than they did at baseline. This corresponds to a doubling to tripling of the distance covered. The reason for this increase may be an improvement in the strength endurance of the muscles involved, an increase in cardiorespiratory endurance, an improvement in the handling of the device, and an improvement in the coordination of the movement that propels the device. These individual components were not analyzed separately in the present study but were only considered in the resulting overall activity of the riding performance. It would be promising to investigate changes in strength endurance through strength testing and cardiopulmonary endurance through VO_2peak_ (peak oxygen consumption) measurements in future studies. Previous studies in children and adolescents with CP have found that cardiorespiratory training can effectively increase cardiorespiratory endurance. The exercise prescription for this population includes a minimum frequency of two to three sessions per week; an intensity between 60% and 95% of the peak heart rate, between 40% and 80% of the heart rate reserve, or between 50% and 65% of VO_2peak_; and a minimum duration of 20 min per session for at least eight consecutive weeks when exercising three times per week or for 16 consecutive weeks when exercising twice per week [34].

When analyzing the health-related quality of life using the PedsQL questionnaire, only the self-reported dimension of emotional functioning showed a significant change. However, the mean difference of 8.89 is in the order of magnitude of the SEM (standard error of the mean) 8.94 [35]. This means that the observed change is unlikely to be greater than what would be expected based on random variance. Although no effects on health-related quality of life were objectified in this study, this effect cannot be ruled out. The short duration of nine weeks may not have been sufficient to cause significant changes. In addition, the use of the devices was limited to the training sessions in the study. This means that the full potential of the devices could not be exploited. The participatory application possibilities of the GTK^®^ would be particularly evident if it were used outside of the study setting. It is likely that it is precisely this use that could have a positive impact on quality of life, both through the participatory aspect and through increased regular physical activity as a result of intrinsic motivation. The GTK^®^ enables children and adolescents with mobility impairments to participate in cycling, a widespread activity in society. It also promotes their independence and allows them to participate in outings and play with their peers. If these motivating aspects lead to regular use of the device, this will promote an increase in physical activity. Regular physical activity is a desirable goal, especially in a population group that has few opportunities to participate independently in sporting activities. A sustained increase in physical activity is associated with various physical and mental health benefits [17,36]. Future research on GTK^®^ could investigate its participatory aspects using qualitative methods.

The present study showed that regular GTK^®^ training is feasible in the population studied. The participants’ motivation for the training sessions was rated by the supervising physiotherapists between moderate and high. Most physiotherapists found the equipment user-friendly and the handling simple and self-explanatory. Many of them appreciated the simultaneous use of arms and legs and the alternating rather than the usual parallel movement of the arms. There are only a few changes to the device itself that the therapists would like to see in the future. A gear shift that the children and adolescents can operate themselves and fewer restrictions in the field of vision were mentioned several times. Although the steering was a challenge at first, all participants have learned to operate it. Therefore, no adjustments are considered necessary.

The limitations of this study lie in the heterogeneity and size of the sample. No generalizable conclusions can be drawn from the results. Further limitations to the validity of this study are the lack of a control group and the fact that the walking tests were only conducted once for each condition, which means that the results are more susceptible to chance. The heterogeneity of the sample was not only a limitation but also a strength in terms of investigating the applicability of the GTK^®^ in a diverse user group. The fact that this study was conducted in the participants’ natural environment also contributed positively to statements about feasibility. Feedback on the usability and user-friendliness of the devices was not only obtained from the patients but also from the therapists. Overall, the results of the present study provide direction for future research.

## 5. Conclusions

The present study investigated the effects of regular GTK^®^ training on walking ability, GTK^®^ driving performance, and health-related quality of life in children and adolescents with walking impairments. In addition, the usability of the devices and the feasibility of regular GTK^®^ training were assessed. While significant short-term improvements in functional walking ability were observed immediately after the GTK^®^ sessions, no long-term effects on functional walking ability or walking endurance were found. Driving performance improved significantly over the training period, suggesting benefits in muscular endurance, cardiorespiratory endurance, equipment handling, and coordination. However, these functional outcome parameters were not examined individually in the present study. With regard to health-related quality of life, no significant change was found in the domain of self-reported emotional function. Regular GTK^®^ training proved to be feasible in practice. It facilitates participation in cycling and promotes independence among children and adolescents with mobility impairments. The long-term impact on functional outcomes, participation, and quality of life should be further investigated, especially considering the potential to increase physical activity and improve overall well-being in this population.

## Figures and Tables

**Table 1:** Characteristics of the study population.

Age	Sex	Diagnosis	Clinical Symptoms	Walking Aids *	Functional Mobility Scale (FMS) ^1^
13	Male	Myelomeningocele	Leg affected by paraparesis	Posterior walker, foot orthotics	2 1 1
16	Female	Spastic bilateral CP (GMFCS ^2^ II)	Leg and arm affected by spastic paraparesis	Walker, AFO ^3^	4 2 1
12	Female	Spastic bilateral CP (GMFCS III)	Leg and arm affected by spastic paraparesis	Posterior walker, AFO	2 2 1
8	Male	Brain-lung-thyroid syndrome	Chorea and ataxia	Posterior walker, AFO	2 1 1
17	Female	ARSACS	Ataxia	None	5 5 1
7	Female	Leukoencephalopathy	Leg affected by spastic paraparesis	AFO	5 5 1
11	Female	Myelomeningocele	Leg affected by paraparesis	AFO	5 5 1
16	Female	Spastic bilateral CP (GMFCS2 II)	Leg affected by spastic paraparesis	None	5 3 1
17	Male	Spastic dyskinetic bilateral CP (GMFCS II)	Leg and arm affected by spastic dyskinetic movement disorder	None	5 5 1

* In this table, only the walking aids used by the participants in the walking tests of the study are listed. ^1^ FSM: Assesses walking ability on a scale of 1 to 6 over three distances, 5, 50 and 500 m. These distances represent the child’s mobility at home, at school, and in public [30]. ^2^ GMFCS: Gross Motor Function Classification System, which is used to categorize children and adolescents with cerebral palsy into 5 different levels based on their gross motor skills [31]. ^3^ AFO: Ankle Foot Orthosis.

**Table 2:** Results of walking ability and GTK^®^ riding performance tests.

TUG	TUG	2MWT	2MWT	6MCT	6MCT	6MCT	6MCT
(s)	(s)	(m)	(m)	(m)	(m)	(n Help-Interventions)	(n Help-Interventions)
BLM	PIM t1	BLM	PIM t1	BLM	PIM	BLM	PIM
57	53	40	42	180	570	3	0
16	22	105	105	110	700	1	0
30	34	80	64	0 *	290	NA *	5
26	36	51	60	140	710	7	0
8	8	151	153	600	950	0	0
12	14	80	103	130	390	2	4
12	11	85	117	200	690	0	0
10	10	132	113	150	700	4	2
10	11	72	140	250	720	2	0
Wilcoxon signed-rank test							
TUG *p* = 0.2033			2MWT *p* = 0.2334			6MCT *p* = 0.003906	

TUG: timed up and go; 2MWT: two-minute walk test; 6MCT: six-minute cycling test; BLM: baseline measurement; PIM: post-intervention measurement; PIM t1: walking tests during post-intervention measurement before GTK^®^ riding session. * Participant not able to operate GTK^®^ despite help-interventions.

**Table 3:** Results of the walking tests of the post-intervention measurement.

TUG (s)	TUG (s)	2MWT (m)	2MWT (m)
PIM t1	PIM t2	PIM t1	PIM t2
53	48	42	53
22	17	105	118
34	31	64	60
36	32	60	48
8	7	153	157
14	12	103	115
11	9	117	90
10	9	113	147
11	10	140	150
Wilcoxon signed-rank test			
TUG *p* = 0.008789		2MWT *p* = 0.4061	

TUG: timed up and go; 2MWT: two-minute walk test; PIM t1: walking tests during post-intervention measurement before GTK^®^ riding session; PIM t2: walking tests during post-intervention measurement after GTK^®^ riding session.

## Data Availability

The raw data supporting the conclusions of this article will be made available by the authors on request due to the small sample size the specific characteristics of the participants, the information is not given publicly because an identification of participants might be possible (data protection regulations).

## Appendix A

**Table A1:** Training parameters of each training session of every study participant.

Participant		1	2	3	4	5	6	7	8	9
Session 1	Duration (min)	27	25	13	24	23	13	31	9	10
	Distance (km)	1.8	1.47	0.62	1.34	2.69	0.8	1.12	0.54	0.49
	Velocity av. (km/h)	4	3.5	2.7	3.3	6.8	3.5	2.3	3.6	3
	Velocity max. (km/h)	10.1	7.4	8.4	9.1	12.7	10.2	4	6.2	13.1
	OMNI	4	4	NA	5	5	5	0	NA	4
Session 2	Duration (min)	23	22	20	19	16	18	28	14	13
	Distance (km)	3.01	2.9	1.13	1.23	1.78	1.08	1.1	1.7	0.9
	Velocity av. (km/h)	7.6	5.6	3.3	3.8	6.8	3.5	2.4	7.1	4
	Velocity max. (km/h)	12.8	8.47	7.5	9.9	13.7	7.3	NA	11.7	8
	OMNI	6	2	8	4	8	7	10	5	6
Session 3	Duration (min)	14	25	15	19	27	21	11	10	15
	Distance (km)	1.36	1.34	0.82	1.56	3.4	1.67	0.52	0.6	1.42
	Velocity av. (km/h)	5.6	3.1	3.2	4.9	7.5	4.8	2.8	3.3	5.8
	Velocity max. (km/h)	10.3	7.5	7.2	12.7	17.5	11.3	6.8	7.3	11.9
	OMNI	5	7	NA	NA	5	10	3	2	3
Session 4	Duration (min)	22	17	13	13	25	23	32	19	23
	Distance (km)	2.3	1.03	0.77	0.89	3.33	1.6	1.76	1.92	2.15
	Velocity av. (km/h)	6.2	3.5	3.3	4	7.8	4.1	3.2	5.9	5.6
	Velocity max. (km/h)	12.7	7.8	6.7	9.9	17.7	10.2	8.8	19.8	10.1
	OMNI	5	5	5	4	6	5	10	4	5
Session 5	Duration (min)	16	7	17	12	30	24	25	20	15
	Distance (km)	1.89	0.43	1.09	1.04	3.02	1.35	1.75	1.64	1.5
	Velocity av. (km/h)	6.7	3.4	3.8	4.9	7.7	3.3	4.2	4.9	5.9
	Velocity max. (km/h)	12.5	5.6	10.3	8.3	18	9.2	12.4	10.1	14.7
	OMNI	2	4	8	6	4	3	10	5	4
Session 6	Duration (min)	16	14	17	21	27	22	23	25	21
	Distance (km)	1.78	1.09	1.28	1.12	3.62	1.51	1.35	2.39	2.48
	Velocity av. (km/h)	6.7	4.6	4.5	3.2	7.8	4.1	3.4	5.6	7.2
	Velocity max. (km/h)	10.1	8.9	9.6	6.8	16.1	11.1	8	10.1	15.4
	OMNI	3	4	5	5	6	NA	NA	3	6
Session 7	Duration (min)	29	23	22	25	29	26	21	20	15
	Distance (km)	3.49	1.3	1.49	1.59	3.45	1.9	1.72	1.55	1.56
	Velocity av. (km/h)	7.2	3.3	4.1	3.9	7.2	4.4	4.9	4.7	6
	Velocity max. (km/h)	14.9	8	12.5	8	15	12.6	17.8	9.8	9.2
	OMNI	3	4	6	3	6	10	10	6	2
Session 8	Duration (min)	34	25	11	23	27	26	41	15	27
	Distance (km)	4.12	1.64	0.5	1.56	2.67	2.58	2.48	1.33	2.55
	Velocity av. (km/h)	7.3	3.9	2.7	4	6	5.9	3.6	5.4	5.6
	Velocity max. (km/h)	14.8	9.2	5.7	7.8	12.7	12.3	10.3	10.3	15.8
	OMNI	4	7	5	1	6	10	10	6	5
Session 9	Duration (min)	36	25	20	22	25	18	30	35	22
	Distance (km)	4.32	1.95	1.23	1.54	3	1.33	1.95	3.5	2.09
	Velocity av. (km/h)	7.3	4.6	3.6	4.2	7.2	4.3	3.8	6	5.7
	Velocity max. (km/h)	15	11.3	11.3	8	NA	9.5	10.2	NA	13.4
	OMNI	3	8	5	1	5	10	6	7	9
Session 10	Duration (min)	24	24	19	27	29	25	31	16	22
	Distance (km)	2.29	1.82	1.23	1.77	4.3	2.35	2.11	1.96	2.5
	Velocity av. (km/h)	5.7	4.5	3.8	3.9	9	5.5	4.1	7.3	6.6
	Velocity max. (km/h)	14.7	8.3	11.3	8	18.9	11.9	9	13.1	11.5
	OMNI	1	6	5	2	5	10	10	6	5
Session 11	Duration (min)	12	20	11	23	34	17	33	19	17
	Distance (km)	1.16	1.14	0.64	1.74	5.63	1.65	2.54	1.77	3.53
	Velocity av. (km/h)	5.6	3.4	3.5	4.5	9.8	5.7	4.6	5.5	12.5
	Velocity max. (km/h)	12.7	8.7	10.2	9.6	22.9	11.5	10.2	9.2	31.9
	OMNI	1	6	2	3	5	10	10	4	4
Session 12	Duration (min)	31	27	13	23	35	22	22	13	18
	Distance (km)	3.44	2.05	0.8	1.65	5.2	1.99	1.8	1.13	2.01
	Velocity av. (km/h)	6.6	4.5	3.9	4.2	7.7	5.5	4.9	5.5	6.6
	Velocity max. (km/h)	13.8	12.7	8	11.4	NA	13.3	10.3	16.1	11.1
	OMNI	3	6	2	3	5	10	6	4	4
Session 13	Duration (min)	19	25	22		34	16	30	17	19
	Distance (km)	1.91	1.92	1.58		4.21	1.26	2.62	1.88	3.13
	Velocity av. (km/h)	6.1	4.6	4.2		7.4	4.8	5.2	6.8	10
	Velocity max. (km/h)	13.7	10.3	10.9		20.5	10.3	9	13.8	13.7
	OMNI	6	4	NA		6	5	8	3	4
Session 14	Duration (min)	24	29	19		15		22	25	
	Distance (km)	2.6	1.86	1.25		2.18		2.11	NA	
	Velocity av. (km/h)	6.3	3.8	3.9		8.8		5.7	NA	
	Velocity max. (km/h)	16.7	12.6	10.3		30.4		10.6	NA	
	OMNI	5	4	5		5		2	3	
Session 15	Duration (min)		31							
	Distance (km)		2.49							
	Velocity av. (km/h)		4.7							
	Velocity max. (km/h)		10.6							
	OMNI		4							
Session 16	Duration (min)		17							
	Distance (km)		1.02							
	Velocity av. (km/h)		3.5							
	Velocity max. (km/h)		9.2							
	OMNI		7							
Session 17	Duration (min)		16							
	Distance (km)		1.49							
	Velocity av. (km/h)		5.4							
	Velocity max. (km/h)		11							
	OMNI		8							

NA: This information is not available due to technical defects on the bike computer or human error.

**Table A2:** Coded answers to PedsQL questionnaire. The table presents the coded answers to the PedsQL questionnaire, categorized by different functioning dimensions. Color coding is used to differentiate these dimensions: Questions 1–8 (gray) assess physical functioning, Questions 9–13 (blue) assess emotional functioning, Questions 14–18 (green) assess social functioning, and Questions 19–23 (orange) assess school functioning.

	Participant 1				Participant 2				Participant 3				Participant 4				Participant 5				Participant 6				Participant 7				Participant 8				Participant 9			
	BLM		PIM		BLM		PIM		BLM		PIM		BLM		PIM		BLM		PIM		BLM		PIM		BLM		PIM		BLM		PIM		BLM		PIM	
	SR	PR	SR	PR	SR	PR	SR	PR	SR	PR	SR	PR	SR	PR	SR	PR	SR	PR	SR	PR	SR	PR	SR	PR	SR	PR	SR	PR	SR	PR	SR	PR	SR	PR	SR	PR
1	25	0	0	0	100	0	100	0	50	0	50	25	0	25	0	25	50	50	75	25	50	0	50	0	0	25	0	25	50	25	0	50	100	50	100	25
2	0	0	0	0	100	0	100	0	100	0	0	0	0	0	0	25	25	25	50	25	25	0	0	0	50	0	0	0	75	0	50	0	0	0	25	0
3	50	75	75	75	100	0	100	0	50	25	0	0	25	75	75	100	75	50	50	25	0	0	100	0	25	0	0	0	75	75	50	75	25	25	50	25
4	100	0	75	0	50	0	50	0	0	0	50	0	75	0	0	25	50	0	25	25	25	0	50	0	75	0	100	0	0	25	50	25	50	25	50	NA
5	25	0	100	0	0	NA	0	0	75	0	0	25	0	NA	0	NA	100	75	100	50	100	NA	0	0	0	50	50	25	50	0	75	25	50	0	25	25
6	0	25	0	25	100	0	50	0	75	25	75	25	50	NA	0	NA	75	50	100	50	25	0	0	0	NA	50	0	50	25	25	0	25	25	25	50	25
7	100	75	100	100	100	NA	100	100	25	50	25	50	100	100	100	25	50	50	50	50	100	NA	100	100	100	75	100	50	25	25	25	75	50	25	0	50
8	100	25	75	75	100	50	75	25	75	50	50	50	50	75	50	50	25	25	0	50	50	50	50	50	0	25	100	25	25	75	50	75	25	50	50	50
9	100	75	100	100	75	100	100	100	50	50	75	50	100	75	100	100	50	50	75	75	100	75	100	75	100	50	100	50	25	25	100	25	50	50	25	100
10	100	NA	100	100	100	100	100	100	50	50	75	50	50	75	100	100	50	50	50	75	100	100	100	100	100	50	100	50	75	50	50	50	25	50	25	75
11	100	NA	75	100	100	100	100	75	50	50	50	50	75	100	100	100	50	50	50	75	50	100	100	100	0	25	0	50	50	75	50	75	25	100	75	100
12	100	100	100	100	100	NA	100	100	50	50	50	50	100	100	100	NA	50	50	25	50	100	100	0	NA	0	75	75	50	50	100	25	100	0	25	25	25
13	100	75	100	75	100	NA	75	100	75	NA	100	NA	100	100	100	100	0	50	0	75	100	100	100	NA	25	75	75	50	25	25	25	0	0	50	25	50
14	100	100	100	100	100	100	100	75	25	50	50	50	50	100	100	100	50	75	50	75	100	100	100	100	100	75	75	75	100	100	75	100	50	NA	50	100
15	100	100	100	100	100	100	100	75	50	75	100	50	75	100	75	100	50	75	50	100	100	100	100	100	75	100	75	100	75	75	75	100	50	NA	75	100
16	100	75	100	100	100	100	100	100	75	75	75	75	75	100	50	100	75	100	75	100	100	100	100	100	0	75	100	100	100	100	100	100	75	NA	100	100
17	100	25	75	25	100	25	100	0	25	25	25	25	75	0	50	NA	25	25	50	25	50	25	100	0	25	50	100	25	50	25	100	50	25	NA	25	25
18	75	25	100	50	100	75	100	100	50	50	50	50	75	100	50	75	25	75	25	25	100	75	50	75	0	50	25	50	50	25	75	25	25	NA	25	50
19	100	25	100	75	100	100	100	75	75	75	75	NA	50	100	100	NA	75	50	50	50	50	100	100	NA	100	50	0	50	100	50	25	50	50	50	0	75
20	75	75	25	100	100	100	100	50	75	50	100	75	25	100	50	NA	25	50	50	75	50	100	100	50	0	50	0	50	50	75	50	75	25	100	25	100
21	100	0	100	100	100	NA	100	NA	50	50	100	NA	50	100	100	NA	50	75	50	100	25	NA	50	NA	100	50	100	50	NA	50	75	50	50	NA	50	NA
22	100	75	75	100	100	100	100	100	50	50	75	75	75	100	50	100	75	50	75	50	75	100	100	100	75	100	100	75	50	25	100	50	100	75	75	100
23	50	100	50	100	100	100	100	100	75	75	100	75	25	50	25	100	50	75	50	50	100	100	75	100	75	75	100	50	50	25	100	75	25	50	50	50
Physical	50	25	53	34	81	8	72	16	56	19	31	22	38	46	28	42	56	41	56	38	47	8	44	19	36	28	44	22	41	31	38	44	41	25	44	29
Emotional	100	83	95	95	95	100	95	95	55	50	70	50	85	90	100	100	40	50	40	70	90	95	80	92	45	55	70	50	45	55	50	50	20	55	35	70
Social	95	65	95	75	100	80	100	70	45	55	60	50	70	80	65	94	45	70	50	65	90	80	90	75	40	70	75	70	75	65	85	75	45	NA	55	75
School	85	55	70	95	100	100	100	81	65	60	90	75	45	90	65	NA	55	60	55	65	60	100	85	83	70	65	60	55	63	45	70	60	50	69	40	81
Total	78	50	75	70	92	64	89	58	55	42	59	43	57	75	60	77	50	53	51	57	68	66	71	55	47	51	60	46	53	47	58	55	39	44	43	60

BLM: Baseline measurement, PIM: Post-intervention measurement, SR: Self-reported, PR: Proxy-reported, NA: No answer.

**Table A3:** Questionnaire on feasibility and usability of GTK^®^ in practice.

**Feasibility and Usability of GTK^®^ in Practice—Questionnaire for Physiotherapists**	
How was the handling of the GTK^®^ for you as a therapist? (preparation, settings, etc.)	- User-friendly and convenient - Practical for getting in (transfer from wheelchair to seat) - The opening was partially blocking - It was easy to prepare as there was little, I needed to change - The GTKs^®^ are heavy and not easy to move when you are not sitting in them - It was quite intuitive in the handling - In the beginning it was a little complicated but by the time it became easier - Very easy if there was not a lot to change - Okay, I understood it - The child was able to instruct me - Sometimes a little tricky to adjust
How was the use of the GTK^®^ for the children and adolescents? (transfers, need for support, steering, gear shifting, etc.)	- Transfer: Pretty self-explanatory and when it was well open - Good handling for getting in and out - The transfers to the GTK^®^ were often unproblematic, but almost all children needed support to position their legs - Transfers from the GTK^®^ to the wheelchair or to standing were difficult and most children needed support - Steering was difficult at first, especially indoors where it is narrower. Most students learned to maneuver after a few training sessions. - The fact that the GTKs^®^ do not have a reverse gear made things more difficult - It was a shame that the children could not change gear themselves. Most of them could have learned how to do it. - The children quickly learned how to brake - Child had quite a bit of trouble making the turn to one side and driving straight ahead - I operated the gears - The seat was a little too big for the child - Not practical that the children could not change gears themselves - Easily feasible transfers - Gear shifting would be better for some children if they had access themselves - The view is restricted by the large part at the front - Transfer inconvenient - The steering needs familiarization - Transfers went well - Child could not operate the gearshift independently while riding
What adjustments would you like to see in a new model?	- Faster adjustment options, such as the distance to the pedals and handlebars - Gear shifting on handlebars - Possibility to quickly adjust the position of the pedals - Integrate reverse gear - Allow the height of the handlebars to be adjusted - Integrate a different tube system on the handlebars (some children have hurt themselves slightly on the inside of their legs or knees) - An invention which helps to make the turn on one side or to drive straight ahead when a unilateral CP is present - A device that allows children with impaired gripping function to cycle with their upper arms without a handle - Front gear shift - Reverse gear so that children can reverse and maneuver independently in tight bends - Gear shift at the front so that children and adolescents can operate it independently (and at the rear for assistance) - Less material in front of the head - Gear shift closer to the steering wheel - Different operation, e.g., larger levers - Less weight overall
Which patient groups do you think the GTK^®^ is suitable for?	- CP leg accentuated (and CP quadriplegic) - People with mild ataxia - Paraplegics and some quadriplegics - Muscle dystrophies rather early stage - Patients who can initiate movement in the upper and lower extremities and have sufficient cognitive and coordination skills to control the GTK^®^ - CP and Spina Bifida - All patients - Patients who can make the transfer with little help, hand motor skills should not be too limited - CP - Paraplegics with low levels of lesion
Who is the GTK^®^ not suitable for?	- Patients with hemiplegia with severe side difference - CP with severe spasticity - Muscle dystrophies at late stage - Children who cannot initiate dissociated movements of the legs or arms - Children with severe upper limb contractures - Children with very limited cognitive abilities - Possibly Spina Bifida - Excessive spasticity of the arm flexors or hip adductors - In the case of muscular dystrophies, the transfer back into the wheelchair is somewhat more complex - Children with major visual impairments - Heavy people with little mobility (transfer) - Patients with ataxia, tetraplegia, hemiplegia
What makes the GTK^®^ different from other tricycles you know? What are the advantages/disadvantages of the GTK^®^ compared to other cycles?	- Simultaneous mobilization of upper and lower extremities - Probably with good effect on tone regulation and activation of the entire body circulation - Advantage: All extremities are trained and used at the same time, which also requires core stability - Disadvantage: Requires a lot of coordination and cognitive skills, excludes many children and cannot be used with very young children - It appears faster and lighter - It needs a bit of space to turn - The steering - Advantages: Comfortable seat - Disadvantages: Poor sight due to the hand pedals in the field of vision - Advantage: Simultaneous arm and leg function - Disadvantage: Coordinatively more difficult to steer - The steering is different - The alternating drive with the arms is good
What improvements have you seen in the children and adolescents as a result of GTK^®^ training?	- Skill in handling the bikes - Increasing endurance - Short-term reduction of spasticity - Learning a new activity/better steering skills and generally better use of the GTK^®^ - Better intermuscular coordination - They could steer it better - Increased endurance - Coordinative improvements through simultaneous arm and leg movements - Endurance training - Steering very difficult at the beginning, familiarization after 4–5 times - Transfers went faster by the time - Speed of movement and the distance became faster and further with practice
How highly do you rate the students’ motivation for the GTK^®^ training? (from 1–10)	
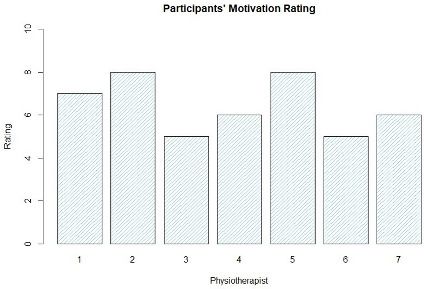

Physiotherapists’ responses on the feasibility of GTK^®^ training and the usability of the GTK^®^ devices in practice, grouped by person.
